# Poly(vinyl alcohol) Hydrogels for Osteoarthritis: A Review of Preparation Strategies, Modification Approaches, and Challenges

**DOI:** 10.3390/gels12060498

**Published:** 2026-06-03

**Authors:** Jiaxuan Di, Yan He, Chao Sun, Jingna Jia, Xing Zheng, Xinyu Li

**Affiliations:** 1 Department of Polymer Materials & Engineering, College of Engineering, Yanbian University, Yanji 133002, China; 1234023183@ybu.edu.cn; 2 Jilin Collaborative Innovation Center for Antibody Engineering, Jilin Medical University, Jilin 132013, China; hy0725@jlmu.edu.cn; 3 Department of Physics, Jilin University, Changchun 130012, China; sunc344@jlu.edu.cn; 4 Normal College, Yanbian University, Yanji 133002, China; 5 Department of Chemistry, College of Science, Yanbian University, Yanji 133002, China

**Keywords:** poly(vinyl alcohol)-based hydrogels, preparation methods, functional modification, cartilage repair

## Abstract

Articular cartilage has attracted significant attention for its essential roles in joint lubrication and stress buffering. However, its inherent self-repair capacity is limited. Addressing inflammatory damage to this tissue, therefore, presents a major clinical challenge in orthopedics. Poly(vinyl alcohol) (PVA)-based hydrogels have emerged as promising repair materials due to their high water content, which mimics the properties of natural cartilage, as well as their tunable mechanical properties and favorable biocompatibility. This review comprehensively examines PVA-based hydrogels, beginning with an overview of their network formation. It then systematically summarizes the main methods and principles for constructing their networks, including physical crosslinking (e.g., cyclic freezing-thawing), chemical crosslinking, and radiation crosslinking, as well as targeted strategies to enhance performance and modify functionality. Particular emphasis is placed on their diverse clinical applications in treating osteoarthritis, primarily including their use as surgical adjuncts, such as injectable gels and anti-adhesion membranes, as long-term or biodegradable cartilage replacement implants, and their potential in partial joint surface resurfacing and reconstruction. Finally, prospects for the application of PVA-based hydrogels in osteoarthritis therapy are considered. Overall, as versatile platform materials, PVA-based hydrogels demonstrate significant potential for clinical translation in cartilage repair.

## 1. Introduction

Articular cartilage defects represent a common clinical problem in modern medicine. Articular cartilage is a vascular and aneural hyaline connective tissue covering the bone ends. It plays a crucial role in load transmission and shock absorption, providing low-friction lubrication during joint movement [1]. Once damaged, however, its intrinsic capacity for self-repair is extremely limited [2]. Small, full-thickness defects (typically <2 mm in diameter) can undergo partial repair by bone marrow mesenchymal stem cells from the subchondral bone. However, the newly formed tissue is typically fibrocartilage, which has poor mechanical properties and is insufficient to withstand long-term joint loading [3]. For larger defects, conventional clinical treatments such as microfracture and mosaicplasty can provide short-term symptomatic relief. Nevertheless, their long-term outcomes are often unpredictable, and the repaired tissue is prone to degeneration [4]. Consequently, there is an urgent need in orthopedics and biomaterials science to develop biomaterials capable of achieving long-term, effective repair or replacement of damaged cartilage [5,6].

Among various cartilage substitute materials, hydrogels are among the most promising. Their advantages include high water content, tunable mechanical properties, and a microenvironment resembling the natural extracellular matrix of cartilage [7]. In our previous studies, cellulose-based hydrogels have served as excellent drug carriers [8]. Moreover, hydrogels can function as acellular fillers for tissue defects, providing mechanical support and facilitating a smooth joint surface [9]. They can also serve as cell carriers for constructing tissue-engineered cartilage, which is implanted to regenerate functional hyaline cartilage [10]. An ideal hydrogel for cartilage replacement must possess a compressive modulus, elasticity, and fatigue resistance matching that of natural cartilage. Furthermore, it requires an extremely low friction coefficient, good wear resistance, and excellent biocompatibility to ensure long-term functional stability within the complex biomechanical environment of the joint cavity [11].

Among hydrogel materials, poly(vinyl alcohol) (PVA) hydrogels, particularly those prepared by physical crosslinking methods such as cyclic freezing-thawing, have shown unique advantages for articular cartilage replacement. Their three-dimensional network structure exhibits mechanical behavior similar to that of the proteoglycan-collagen network in natural cartilage, providing effective support and good biocompatibility [12]. HA hydrogels exhibit low mechanical strength (only tens to hundreds of kPa), making them unable to withstand joint loading, and they degrade rapidly within days to weeks in vivo. In contrast, through cyclic freeze–thaw processing, PVA parameters such as concentration, molecular weight, and crosslinking mode can be precisely controlled to achieve a compressive modulus matching that of natural cartilage (0.5–2 MPa), along with excellent fatigue resistance and long-term stability, maintaining integrity in vivo for several months or more. The friction coefficients of the two materials are comparable (approximately 0.02); the non-degradable nature of PVA in this application provides durable, stable mechanical support, thereby avoiding the inflammatory response triggered by HA degradation [13]. However, the biological inertness and non-degradability of PVA also limit its application in promoting cell ingrowth and functional tissue regeneration. In recent years, several reviews have systematically summarized the applications of hydrogels in cartilage repair and poly(vinyl alcohol) (PVA)-based hydrogels in the biomedical field. However, most studies merely introduce physical, chemical, or radiation crosslinking methods in isolation, lacking a consistent comparative framework. In particular, few reviews have discussed in detail the differences among these methods in terms of scalability, biosafety, fatigue life under load-bearing joint conditions, and their specific applications in osteoarthritis treatment as surgical adjunct materials, alternative implants, and surface replacement or reconstruction. Yet these factors are precisely the key determinants of whether laboratory outcomes can ultimately be translated into clinical practice. Therefore, the core of current research lies in functionally modifying PVA to construct multifunctional, integrated PVA-based hydrogel systems, aiming to transition from “passive replacement” to “active regeneration.”

## 2. Strategies for the Preparation and Functionalization of PVA

The preparation and functionalization strategies for poly(vinyl alcohol) (PVA) are evolving from traditional single-network construction towards highly customized, intelligent, and functionally integrated multi-purpose systems [14]. For PVA-based hydrogels intended for intra-articular administration, priority should be given to rheological properties similar to those of healthy synovial fluid, exhibiting shear-thinning non-Newtonian behavior. They must possess mechanical strength matching that of natural cartilage, with a typical Young’s modulus in the range of 200–5400 kPa to bear physiological loads. At the same time, long-term stable low-friction lubrication is required, with a coefficient of friction close to or below 0.05 to avoid wear of the opposing cartilage, along with good thixotropy and injectability [15]. With the deep integration of materials science and biomedicine, future PVA-based materials are envisioned not merely as bioinert scaffolds. Instead, they are emerging as new biomaterials capable of actively sensing physiological signals, dynamically adjusting, enabling targeted therapy, and ultimately undergoing safe metabolism [16]. To this end, Table 1 systematically summarizes the core features of three mainstream strategies used for the preparation and functionalization of PVA. This overview aims to provide new directions for the development of PVA-based materials.

### 2.1. Network Construction Methods

Constructing a stable three-dimensional network structure is the fundamental step in preparing hydrogels [17]. As summarized in Table 2, the primary methods are physical, chemical, and radiation crosslinking. Physical crosslinking, particularly the cyclic freezing-thawing method, is widely used for its mild process and the absence of chemical additives [18].

In 2022, Adelnia et al. [19] presented a comprehensive review of polyvinyl alcohol (PVA) hydrogels prepared by the freeze/thaw (F-T) process. During freezing, PVA polymer chains are expelled into the unfrozen aqueous phase, forming concentrated regions that crystallize. These microcrystalline regions act as physical crosslinking points, conferring significant advantages in mechanical properties (elastic modulus, tensile strength) compared to non-crystallized hydrogels. In 2024, Górska et al. [20] further improved the freeze–thaw process by selecting appropriate polymers as matrix-forming agents and optimizing the freezing temperature, thereby achieving structural integrity in the presence of exudates, adequate mechanical strength, high flexibility, low adhesion, and slightly acidic pH. Advanced analyses using differential scanning calorimetry (DSC) and low-field nuclear magnetic resonance relaxometry (LF TD NMR) were employed to evaluate mechanical properties, absorption capacity, and microstructure. Hassan et al. [21] demonstrated that freezing/thawing-prepared hydrogels possess high water content (>90%) and viscoelastic properties closely resembling those of natural cartilage. These findings established a crucial foundation for subsequent cartilage replacement research.

Chemical crosslinking primarily uses multifunctional small molecules to react with PVA hydroxyl groups, forming covalent networks. In 2014, Kudo et al. [22] investigated the effect of glutaraldehyde, a crosslinker, on the structure and swelling behavior of PVA hydrogels. They found that by controlling the glutaraldehyde concentration and reaction pH, they could obtain hydrogels with tunable swelling ratios and enhanced mechanical strength. The polymer network and water structure varied with gel preparation, crosslinking method, and drying/rehydration processes. However, the potential toxicity of residual aldehyde groups remained a major challenge. In 2010, Sabagh et al. [23] studied the chemical crosslinking of PVA hydrogels with different crosslinkers (e.g., glutaraldehyde and epichlorohydrin) in the presence of a catalyst. Three types of hydrogels were produced. The effects of pH and temperature on the swelling characteristics of these prepared gels were investigated. The study indicated that low concentrations of crosslinkers yielded hydrogels with moderate properties. However, hydrogels obtained without crosslinkers exhibited good performance and could serve as eco-friendly absorbents in organic solvents. Although hydrogel formulations containing hyaluronic acid (HA) as the active ingredient currently occupy the vast majority of the market share on pharmacy shelves, poly(vinyl alcohol) (PVA)-based hydrogel formulations are also commercially available, and the two materials have different focuses in biomedical applications. HA hydrogels, leveraging their inherent moisture-retaining properties, dominate superficial lubrication scenarios, whereas PVA hydrogels—once residual chemical crosslinkers are controlled—are becoming a research hotspot for load-bearing tissue engineering applications such as cartilage repair, owing to their superior mechanical strength and stability.

**Figure 1:**
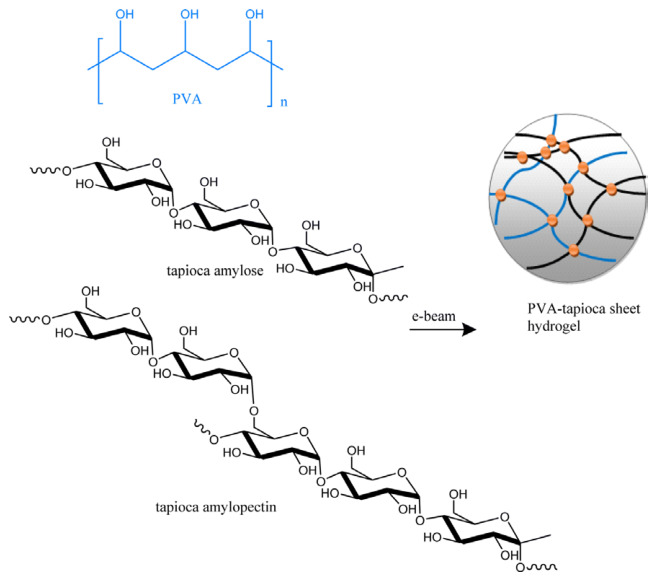
Fabrication of PVA/cassava starch hydrogel via electron beam crosslinking. Reproduced with permission from ref. [27]. Copyright 2024, RSC.

Radiation crosslinking offers another convenient approach. Crosslinking induced by ultraviolet, electron-beam, or gamma-ray irradiation generates free radicals and promotes covalent bond formation between functional groups [24]. In 1999, Rosiak et al. [25] thoroughly investigated the mechanism and kinetics of crosslinking in PVA aqueous solutions initiated by γ-ray radiation. They found that radiation could induce both crosslinking and chain scission. The final network structure depended on the radiation dose and PVA concentration. This method requires no chemical additives and yields pure products. The crosslinking density can be precisely controlled by adjusting the radiation dose, making it highly suitable for preparing high-purity cartilage substitute materials. In 2024, Chen et al. [26] prepared pre-made PVA/CNF hydrogels via gamma-ray irradiation and obtained composite hydrogels by annealing at 80 °C after fixing the hydrogel ends. Experiments showed that, compared with the pristine hydrogel, the crystallinity increased significantly after annealing, and the tensile strength increased dramatically from approximately 65 kPa to over 21 MPa, with an elastic modulus of 4.2 MPa. Meanwhile, the lubrication performance was also improved, demonstrating great potential as an artificial joint cartilage material. Recently, Khalid et al. [27] fabricated PVA/cassava starch hydrogels via electron-beam crosslinking at 25 kGy, as illustrated in Figure 1. They subsequently characterized the surface morphology, gel fraction, swelling ratio, moisture retention capacity, and thermal stability. The resulting hydrogels exhibited optimal porosity, mechanical strength, thermal stability, and moisture retention, making them well-suited for the intended application. In vitro assays demonstrated 90% fibroblast viability, suggesting its potential as a cost-effective wound dressing to accelerate the healing of acid burn injuries.

In the treatment of osteoarthritis, the clinical applicability of the three crosslinking methods varies significantly. Physical crosslinking (freeze–thaw method) offers low cost and absence of additive toxicity, but its insufficient mechanical strength makes it difficult to sustain long-term joint loading. Chemical crosslinking can enhance mechanical properties, yet residual crosslinker cytotoxicity remains a major obstacle, along with harsh reaction conditions and poor batch-to-batch reproducibility. Radiation crosslinking enables the production of high-purity sterile products with precisely controllable crosslinking density; however, the equipment is expensive, and large-scale production is challenging. Overall, physical crosslinking is the most readily translatable approach due to its low cost and high safety profile, although it requires subsequent reinforcement strategies to compensate for its mechanical shortcomings. Radiation crosslinking offers unique advantages in high-purity medical products, whereas chemical crosslinking requires the development of non-toxic crosslinking systems or efficient residue removal processes.

### 2.2. Mechanical Property Enhancement

Meeting the stringent mechanical demands for load-bearing tissue repair requires effective modification of PVA. As shown in Table 3, the mechanical properties of PVA hydrogels vary significantly with different preparation methods. The inherent loose structure of single PVA networks limits their mechanical and biological performance [28].

Interpenetrating polymer networks (IPNs) represent one effective strategy for enhancing structural stability and mechanical properties. In 2003, Gong et al. [29] first generated extremely strong hydrogels (fracture stress reaching tens of MPa) by inducing double-network (DN) structures in various combinations of hydrophilic polymers. In 2014, Pulat et al. [30] synthesized semi-IPN hydrogels with different compositions via free-radical polymerization using ethylene glycol dimethacrylate as the crosslinker. These hydrogels consisted of poly(vinyl alcohol) (PVA), polyacrylate (PAA), and polyacrylamide (PAAm). At pH 7.4 and 37 °C, the most swellable PAA/PVA hydrogel exhibited an average swelling percentage of 1660%, while the least swellable IPN hydrogel (PAAm/PVA) showed 550%. In 2024, Santos et al. [31] innovatively introduced meta-aramid (Nomex^®^) nanostructures into PVA networks. Incorporating 1.5 wt% Nomex^®^ significantly enhanced mechanical properties due to the inherent strength of aramid and hydrogen bonding with PVA chains. Gamma-ray sterilization did not impair material performance; instead, it may have induced additional crosslinking, further enhancing mechanical properties.

**Figure 2:**
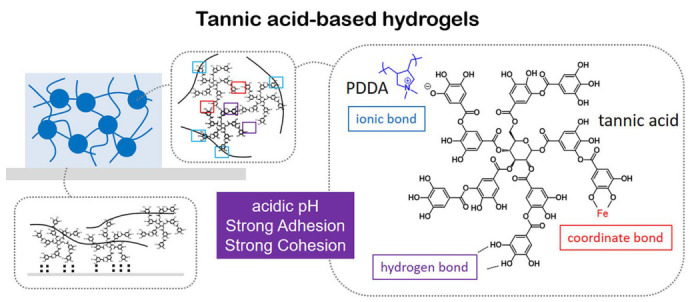
Schematic illustration of the tannic acid-based supramolecular hydrogel. Reproduced with permission from ref. [33]. Copyright 2017, ACS.

Introducing dynamic reversible bonds represents a cutting-edge approach to enhancing PVA hydrogels while imparting smart properties. In 2012, Sun et al. [32] developed ultra-tough, self-healing PVA hydrogels using Fe^3+^ multi-coordination with hydroxyl and catechol groups on PVA chains. These hydrogels maintained 90% water content while exhibiting tensile strength over 20 times the initial value, fracture energy up to 9000 J/m^2^, and rapid room-temperature self-healing after damage. In 2017, Fan et al. [33] introduced tannic acid into PVA networks, leveraging synergistic multiple hydrogen bonds and coordination interactions between tannic acid’s abundant phenolic hydroxyls, PVA chains, and metal ions (e.g., Fe^3+^). The resulting hydrogel, shown in Figure 2, achieved a storage modulus of 0.15 MPa and conductivity of 4.3 S × m^−1^, creating a multifunctional wound dressing with excellent adhesion, antioxidant properties, and high strength. In 2023, Zhang et al. [34] rapidly gelled a dynamic alginate hydrogel through dynamic covalent bonds between phenylboronic acid-modified alginate and poly(vinyl alcohol) (PVA). In vitro studies showed that the bioprinted scaffold could reduce intracellular oxidative stress in encapsulated chondrocytes during H_2_O_2_ exposure and protect them from H_2_O_2_-induced downregulation of extracellular matrix (ECM)-related anabolic genes and upregulation of catabolic genes, demonstrating promising application prospects.

Biomimetic structural design has also inspired mechanical reinforcement strategies. In 2020, Guan et al. [35], inspired by the “brick-and-mortar” structure of nacre, combined PVA with montmorillonite nanosheets via freeze-casting and cyclic freezing-thawing. The resulting nanocomposite exhibited highly ordered layered structures, with strength and toughness tens of times higher than those of pure PVA hydrogels, along with excellent fatigue resistance, offering a new paradigm for the development of artificial cartilage and ligaments. In 2024, Ji et al. [36] innovatively prepared multiscale-reinforced PVA hydrogels by chemically crosslinking polyvinylpyrrolidone (PVP) and in situ assembling aramid nanofibers (ANFs), thereby achieving reinforcement ranging from the molecular level to the nanoscale. The optimized hydrogel exhibited high tensile strength (~10 MPa), high toughness, and an 80% water content, making it promising for applications such as biological tissue replacement, flexible wearables, electronic skin, and in vivo sensors. In 2022, Mredha et al. [37] mimicked wood channel structures by unidirectionally freezing PVA/cellulose nanofiber (CNF) solutions. Ice crystal growth expelled CNF and PVA to grain boundaries, forming highly oriented tubular channels. After thawing, the resulting composite hydrogel exhibited significantly anisotropic mechanical properties and guided cell migration. In 2024, Soto-Quintero et al. [38] simulated the native extracellular matrix by incorporating naturally occurring biopolymers, such as collagen (COL) and hyaluronic acid (HA), into ionically crosslinked poly(vinyl alcohol) (PVA)/sodium alginate (SA) fibrous scaffolds. They found that the presence of these biopolymers not only increased the hydrogen-bonding density but also induced the formation of hydrogen-bonded, crosslinked scaffolds with crystalline PVA domains and well-defined fibrous structures. The results demonstrated that even at low concentrations, the addition of natural macromolecules increased hydrogen-bond density and altered the characteristics of PVA crystals, indicating great potential for cartilage repair.

Addressing the application requirements for load-bearing sites in osteoarthritis treatment, the aforementioned mechanical property enhancement strategies each have advantages and disadvantages regarding long-term performance and clinical scalability. Interpenetrating polymer networks (IPNs) significantly improve mechanical properties and benefit from a relatively mature fabrication process; however, the synthesis method is complex, and long-term fatigue stability has not yet been verified. Dynamic reversible bonds impart self-healing and smart-responsive characteristics, but their long-term stability in physiological environments is poor, and metal ions may induce oxidative stress. Biomimetic structures (e.g., brick-and-mortar layered architectures) exhibit outstanding mechanical performance (with strengths in the several tens of MPa range). Yet they rely on complex templates and freeze-casting techniques, which make large-scale production difficult. Overall, the IPN strategy is closer to clinical practicality in terms of mechanical performance and process maturity, but it still requires process simplification. Dynamic bonds are more suitable for non-load-bearing or low-load scenarios. Although biomimetic structures offer exceptional mechanical properties, their scalability remains the most significant bottleneck at present.

### 2.3. Biofunctionalization

The goal of next-generation PVA-based hydrogels has evolved from “passive replacement” to “active regeneration.” This paradigm shift requires materials not only to fill defects but also to create a microenvironment conducive to host cell migration, proliferation, and eventual formation of new functional cartilage. Although PVA formulations have the weaknesses of being degradable by specific microorganisms and lacking inherent antimicrobial properties, these risks are not uncontrollable. Degradation by specific microorganisms is also a major guideline in our pharmaceutical practice. By adhering to strict pharmacopoeial quality standards, employing aseptic manufacturing processes, and implementing rational preservation strategies, the safety and efficacy of PVA formulations can be fully ensured [39]. Table 4 summarizes common biofunctionalization strategies. Among these, incorporating bioactive molecules (e.g., growth factors and small-molecule drugs) into hydrogels is the most direct approach.

**Figure 3:**
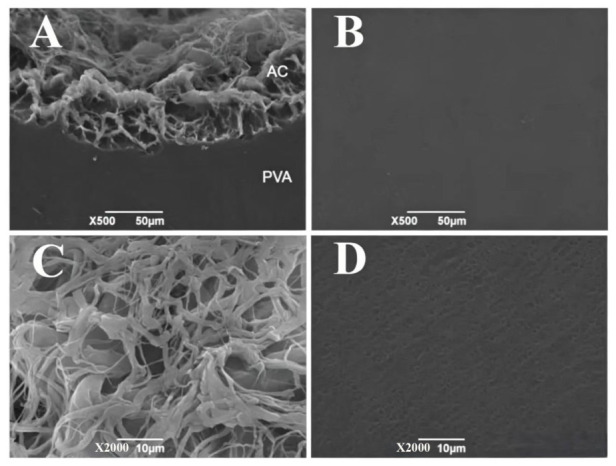
SEM micrographs of the morphology and ultrastructure of PVA/AC bio-hybrid scaffolds. In the PVA/AC_bilayer scaffold, the porous AC layer is in continuous contact with the synthetic PVA layer (**A**); at higher magnification, its interconnected porous network is clearly visible (**C**). In the PVA/AC_blend scaffold, nanoscale pores are less evident due to lower porosity and smaller average pore size, resulting in a more homogeneous surface (**B**,**D**). Reproduced with permission from ref. [43]. Copyright 2025, Frontiers.

Biofunctionalization, through the grafting of anti-inflammatory factors and growth factors, provides the overarching direction for cell recruitment, inflammation suppression, and oriented differentiation in cartilage regeneration. Degradation control determines the rate and timing of signaling molecule release, providing specific spatial cues for mechanical support and tissue growth in nascent tissue. Only through the precise integration of these two aspects can a complete regenerative loop be achieved—from regulation of inflammation to chondrogenic differentiation and ultimately to functional tissue replacement. Specifically, the spatiotemporal controlled release of growth factors is the core technology for functionalizing PVA-based hydrogels for cartilage repair. In 2015, Nie et al. [40] loaded transforming growth factor β1 (TGF-β1) into PVA hydrogels and achieved controlled, phase-dependent promotion of chondrocyte adhesion and proliferation by regulating the release kinetics. The key advantage of this strategy is the stepwise release of growth factors in accordance with the degradation rate, which facilitates cell recruitment at the initial stage and maintains chondrogenic phenotype differentiation at later stages, thereby mimicking the dynamic changes in growth factor concentration during natural cartilage development. In the same year, Hou et al. [41] introduced natural polysaccharides, namely HA and CS, into freeze-thawed PVA and magnetic nanocomposite PVA hydrogels. The presence of the polysaccharides significantly increased the pore size and equilibrium swelling ratio (ESR) of the PVA hydrogels. Experimental results showed that incorporating natural polysaccharides promoted chondrocyte adhesion and proliferation on PVA hydrogels while preserving mechanical strength, demonstrating great potential for cartilage repair. In 2020, Oliveira et al. [42] systematically evaluated the feasibility of loading diclofenac, a non-steroidal anti-inflammatory drug, into PVA hydrogels. They found that PVA materials annealed at 150 °C exhibited optimal mechanical properties, and that the combination of vitamin E and cetalkonium chloride effectively controlled drug release within 24 h without inducing irritation or cytotoxicity. This study provides a dual-function platform that combines mechanical support and drug intervention to control early-stage acute inflammation after cartilage surgery. In 2025, Barbon et al. [43] developed a biohybrid scaffold by combining decellularized human articular cartilage with PVA hydrogel, with bilayer and blended structures as shown in Figure 3. Among these, the bilayer scaffold best supported the adhesion and growth of mesenchymal stem cells. The natural extracellular matrix components in the decellularized cartilage effectively compensated for the lack of bioactive recognition sites in synthetic PVA. Collectively, these studies demonstrate that, whether through the controlled delivery of exogenous growth factors, the local sustained release of small-molecule drugs, or the integration of bioactive components derived from decellularized tissues, conventional PVA hydrogels can be endowed with enhanced tissue remodeling capabilities.

In summary, the process of cell regeneration can be outlined as follows: PVA-based hydrogels enhance cell adhesion and matrix synthesis through the incorporation of peptides or decellularized matrix components; loaded anti-inflammatory factors or exosomes can induce macrophage polarization toward a reparative phenotype, reduce inflammation levels, and create a favorable regenerative microenvironment; appropriate mechanical modulus and viscoelastic properties regulate chondrocyte phenotype and maintain their function; components such as polyphenols, polysaccharides, or nanosilicates in the material scavenge reactive oxygen species and alleviate oxidative stress damage; meanwhile, the degradation rate of the hydrogel is matched to the growth of new tissue, and the sequential release of growth factors or microRNAs guides matrix remodeling, thereby achieving a complete closed loop from inflammation regulation to functional cartilage replacement.

**Figure 4:**
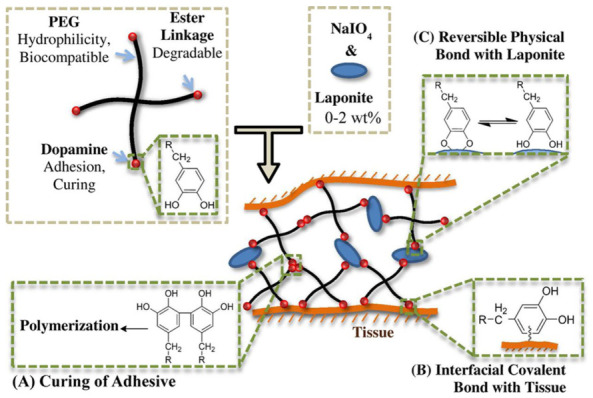
Schematic representation of applying the nanocomposite adhesive to tissue by mixing PEG–D4 with NaIO_4_ and laponite. Reproduced with permission from ref. [46]. Copyright 2021, ACS.

A key challenge in biofunctionalization is achieving long-term, controlled release of these factors to prevent adverse effects from initial burst release and maintain long-term efficacy. In 2020, Wu et al. [44] functionalized poly(vinyl alcohol) (PVA)/polyacrylamide (PAAm) hydrogel surfaces using dopamine (PDA) and the tetrapeptide Arg-Glu-Asp-Val (REDV) to improve cell adhesion. This coating reduced hydrogel hydrophilicity while enhancing protein adsorption, adhesion, viability, proliferation, and spreading of porcine iliac artery endothelial cells (PIECs). The coating also demonstrated favorable hemocompatibility, providing insights into the development of novel hydrogel-based small-diameter vascular grafts. In addition to surface coating modifications, significant progress has also been made in the spatiotemporal functional regulation within the hydrogel network. In 2026, Xie et al. [45] used single-cell RNA sequencing to identify a subpopulation of bone marrow mesenchymal stem cells (CD56^+^CD271^+^). Exosomes derived from these cells were modified with a chondrocyte-specific peptide and loaded into a PVA/sodium alginate composite hydrogel. This system significantly improved cartilage structure in osteoarthritis, enhanced matrix deposition, and suppressed key degenerative and aging-related markers. Mechanistic studies revealed that the therapeutic effect was mediated by exosome-delivered microRNAs inhibiting specific targets in chondrocytes. More notably, in 2014, Liu et al. [46] developed a facile “dip-coating-photocrosslinking” method, as illustrated in Figure 4. Dopamine-modified four-arm polyethylene glycol (PEG-D4) was combined with synthetic nanosilicate laponite to form an injectable, self-recovering tissue-adhesive hydrogel. Modifying PVA hydrogel surfaces with composite coatings containing cell-adhesive peptides and polysaccharides successfully enhanced chondrocyte-specific adhesion and functional expression. In 2026, Li et al. [47] developed a Janus hydrogel with spatiotemporal programming capabilities based on the design concept of “structure-function bionics.” This system constructs a double-network hydrogel structure through the synergistic effects of a PVA-NB covalent network and an HP-PVA dynamic network, utilizing a dynamic borate ester network to achieve high mechanical strength and stress redistribution. Meanwhile, leveraging the principle of polarity-driven migration, a Janus structure with asymmetric adhesion was fabricated, enabling rapid antibacterial activity and long-term inhibition of drug-resistant bacteria while undergoing pH-responsive release during the tissue repair process.

**Figure 5:**
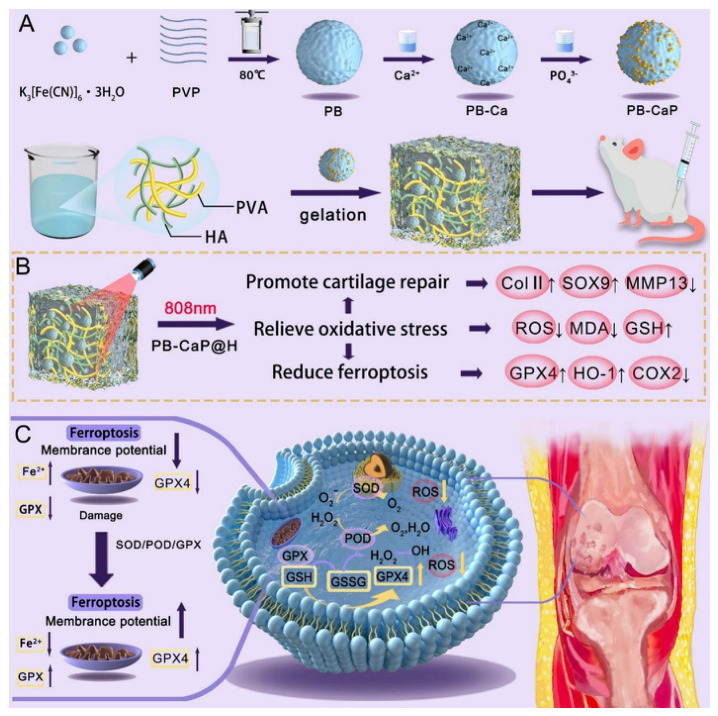
The structure of the PB-CaP nanozyme-laden hydrogel (PB-CaP@H) and its application in treating osteoarthritis. Under near-infrared illumination, the injected PB-CaP@H effectively scavenges reactive oxygen species (ROS) in chondrocytes and repairs damaged mitochondria. Reproduced with permission from ref. [54]. Copyright 2025, Elsevier.

For controlled degradation of PVA, introducing degradable units into the material is the most common strategy. However, PVA itself exhibits poor biodegradability under physiological conditions, primarily due to its stable C–C backbone and high crystallinity, which limit hydrolytic or enzymatic cleavage. As a result, its degradation time (ranging from months to years) often does not match the period required for cartilage regeneration (weeks to months) [48,49]. To overcome these intrinsic limitations, in 2002, Cai et al. [50] developed a biodegradable material by blending polylactide (PLA) with naturally degradable dextran. A novel sponge-like scaffold was fabricated using solvent casting and particulate leaching techniques. After removal of TMS groups, PLA-dextran blend films exhibited significantly enhanced surface and bulk hydrophilicity compared to PLA alone. Cell culture on these polymer substrates showed improved cell attachment and proliferation. Integrating such strategies with PVA hydrogels could achieve tunable degradation ranging from weeks to months. In 2019, Deller et al. [51] successfully immobilized thrombin-polymer surfactant complexes on mesenchymal stem cell membranes. This configuration enabled cell surface-bound thrombin to catalyze local fibrin hydrogel formation, effectively encapsulating cells while maintaining stemness and supporting proliferation and differentiation. Integrating this approach with hydrogels to support cartilage could similarly promote cell-mediated matrix formation. In addition to the design of enzyme-responsive degradation chemistry, research into regulating PVA degradation behavior by incorporating bioactive components is advancing rapidly. To address the lack of enzyme-cleavable sites in pure PVA networks, in 2024, Thai et al. [52] developed a chitosan–poly(vinyl alcohol) methacrylate hybrid hydrogel platform. By employing two different photoinitiators and adjusting the polymer content and photoinitiator types, they achieved independent control over the degradation rate and mechanical properties, providing a feasible engineering strategy to address the core challenge in cartilage tissue engineering—matching the degradation rate with the rate of tissue neogenesis. In 2025, Hu et al. [53] systematically investigated the integration of enzymes into hydrogels. By introducing enzyme-cleavable peptide segments as crosslinkers into PVA networks, they constructed enzyme-responsive degradable hydrogels. This “smart” gel’s selective degradation capability lies at the foundation for constructing dynamic scaffolds that respond to the tissue-regeneration microenvironment. Furthermore, to combat the oxidative stress that often hinders timely degradation and tissue integration, in the same year, Hu et al. [54] prepared a PVA-HA hydrogel incorporating PB-CaP into the matrix, as shown in Figure 5. Experimental evidence showed that the intrinsic enzyme-like activities of PB helped scavenge reactive oxygen species and alleviate oxidative stress. Meanwhile, the nanozyme restored the damaged mitochondrial membrane potential and reduced iron overload in chondrocytes via calcium-ion coordination. Molecular biology studies revealed that the hydrogel upregulated cartilage-related proteins and downregulated degradative enzymes, thereby promoting chondrocyte migration and proliferation. In summary, strategies for controlling the degradation behavior of PVA-based hydrogels have evolved from early single-chemical modifications to multi-level synergistic regulation, encompassing innovative routes such as component blending, hybrid networks, dual-network enzymatic mineralization, and multifunctional coating integration [55]. These approaches collectively tackle the fundamental limitations of PVA—slow hydrolysis, lack of enzymatic recognition, and uncontrolled degradation—by introducing biodegradable co-polymers, enzyme-cleavable moieties, and bioactive nanozymes. The common goal is to achieve dynamic matching between the degradation profile of PVA materials and the process of cartilage tissue regeneration [56,57].

Surface modification is simple to operate and low-cost, but the coating’s long-term stability is poor, making it difficult to achieve spatiotemporally controlled release of active molecules. Growth factor loading offers a clear pro-regenerative effect; however, it suffers from high cost, rapid deactivation, a high risk of burst release, and significant batch-to-batch variability. Degradable units (e.g., enzyme-cleavable peptides) enable dynamic matching, but patient-to-patient differences in microenvironments render degradation rates unpredictable, and long-term safety data remain insufficient. Emerging strategies such as exosomes or nanozymes show great potential, yet their separation and purification costs are high, and clinical translation remains distant. For osteoarthritis, a chronic disease, an ideal PVA hydrogel should balance cost-effectiveness, long-term mechanical stability, and dynamic bioactivity. In the future, combinatorial strategies (e.g., physical crosslinking + biomimetic structures + exosome loading) may enable clinical translation.

## 3. PVA Hydrogels for Articular Cartilage Repair

Articular cartilage repair presents multi-level clinical challenges, spanning from localized defects to complete tissue necrosis. As summarized in Table 5, poly(vinyl alcohol)-based hydrogels—with their excellent mechanical properties, biocompatibility, and functionalizability—have enabled the development of diverse materials tailored to different repair objectives and surgical scenarios. These approaches collectively realize a comprehensive repair logic spanning micro-scale regeneration induction, intraoperative assistance, and macro-scale mechanical replacement, demonstrating the broad application prospects of PVA hydrogels in this field. However, most PVA-based hydrogels are still at the in vitro and preclinical research stages, lacking clinical study experience.

### 3.1. Surgical Adjunctive Materials

In cartilage repair surgeries (e.g., microfracture, cartilage transplantation), PVA hydrogels also serve as adjunctive materials to improve the local surgical environment and prevent complications, thereby enhancing overall repair success rates. Their primary applications include post-operative anti-adhesion barriers and local drug/cell delivery vehicles.

Post-operative adhesion prevention remains a common challenge in orthopedic surgery. Within joints, particularly in the patellofemoral joint or at post-surgical wound sites, fibrous adhesions may form between tissues, leading to joint stiffness and pain [58]. PVA hydrogel films or sprayable gels can serve as ideal physical barriers. In 2005, Liu et al. [59] evaluated the efficacy of PVA hydrogel films in a rat abdominal adhesion model, confirming their ability to effectively isolate tissue surfaces. Combining PVA with hyaluronic acid (HA), they demonstrated that a HA gel containing 0.625% mitomycin C (MMC) was most effective at reducing postoperative adhesion formation. In 2014, Bae et al. [60] tested various PVA/gelatin-based hydrogel compositions for anti-adhesion properties. Rat model experiments showed that PVA/gelatin films (10/90 ratio) significantly reduced the extent of adhesion and were confirmed as potential candidates for anti-adhesion applications. In 2024, Ma et al. [61] designed a novel super-structured porous hydrogel (PVA/PAAc-N) using a moisture-induced phase separation-solvent exchange process, with a porous PVA hydrogel serving as the dissipation layer. This hydrogel exhibited unique ultra-low swelling (0.29) and effectively repaired ruptured porcine hearts, while the PVA surface layer prevented post-operative adhesion, demonstrating significant potential for tissue repair. Through preclinical experiments, this “barrier + therapy” design concept transforms PVA hydrogels from passive barriers into active modulators.

**Figure 6:**
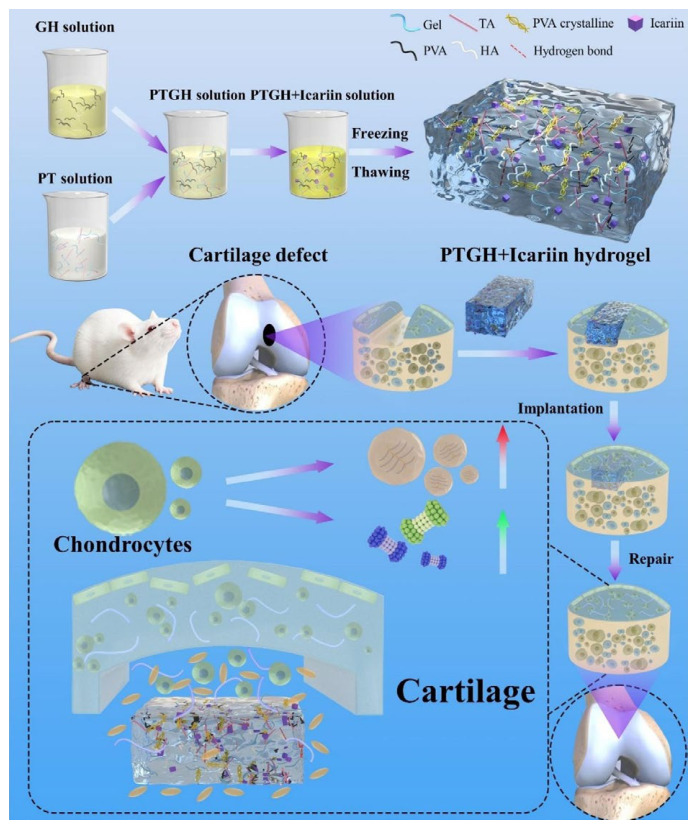
Schematic illustration of PTGH + Icariin hydrogel preparation. Reproduced with permission from ref. [67]. Copyright 2024, Elsevier.

Beyond physical isolation, PVA hydrogels can function as localized delivery platforms for drugs, growth factors, or cells, establishing a sustained-release therapeutic microenvironment at the surgical site to prevent complications. This requires ensuring pseudoplasticity and an appropriate viscosity in the gel. By adjusting parameters such as the crosslinking density and molecular weight of the hydrogel, the compactness of its microscopic network structure can be altered, thereby controlling the drug release rate [62]. Cartilage repair often requires delivery of anti-inflammatory drugs (e.g., diclofenac sodium), analgesics, or cartilage regeneration-promoting growth factors (e.g., TGF-β, BMP-2) [63]. Compared to systemic administration, localized delivery significantly increases drug concentration at the lesion site while reducing systemic toxicity [64]. In 2012, Spiller et al. [65] encapsulated IGF-1, an important growth factor in cartilage regeneration, within biodegradable poly(lactic-co-glycolic acid) (PLGA) microparticles embedded in PVA hydrogels. Murine in vivo studies demonstrated that IGF-1 release promoted chondrogenesis in the hydrogel’s surrounding layer and improved integration between cartilage and hydrogel. The compressive modulus of cartilage-hydrogel constructs without IGF-1 was 0.07 ± 0.02 MPa, compared to 0.17–0.2 MPa for IGF-1-containing hydrogels. This study demonstrated that sustained IGF-1 release promotes tissue formation and provides new directions for cartilage integration with surrounding tissues. In 2015, Dashtdar et al. [66] investigated whether mesenchymal stem cells (MSCs) implanted in novel PVA-chitosan composite hydrogels could improve cartilage repair outcomes in rabbit medial femoral condyles. Results showed significantly higher hyaline cartilage proportions, glycosaminoglycan content, and mechanical properties in the treatment group compared with microfracture alone, with therapeutic effects comparable to those of previously established alginate-MSC constructs. In 2024, Xiang et al. [67] prepared a PTGH + Icariin hydrogel, shown in Figure 6, from poly(vinyl alcohol) (PVA), tannic acid (TA), gelatin (Gel), hyaluronic acid (HA), and icariin. This hydrogel effectively absorbed exudate in mouse defect areas and promoted cartilage defect healing. The PTGH + Icariin hydrogel degraded rapidly initially, then gradually stabilized, providing space for new tissue growth during early cartilage regeneration. The compressive strength (2.87 ± 0.45 MPa) and compressive modulus (0.761 ± 0.072 MPa) of the PTGH + Icariin group were significantly higher than those of other groups, demonstrating that this hydrogel promotes cartilage regeneration and improves mechanical properties in defect areas. The above experiments indicate that PVA-based hydrogels, serving as local controlled-release platforms, can both provide mechanical support and actively guide tissue regeneration through the sustained delivery of bioactive molecules or cells, demonstrating clear application potential in cartilage repair.

As surgical adjunctive materials, the successful application of PVA hydrogels critically depends on matching their biodegradability with functional duration. These materials have evolved from simple physical anti-adhesion membranes into multifunctional intelligent systems integrating isolation, anti-inflammatory, antibacterial, analgesic, and pro-regenerative capabilities, with functionality becoming increasingly sophisticated [68]. However, attention must be paid to timely degradation and absorption after these materials fulfill their mission (e.g., covering the critical window period for tissue healing) to avoid complications from long-term retention. Therefore, controlling the composite ratio of PVA with degradable components, such as gelatin and hyaluronic acid, is a key technology for achieving ideal surgical adjunctive materials.

### 3.2. Replacement Implants

For localized cartilage defects resulting from trauma or early degeneration, directly utilizing PVA hydrogels as filling or replacement implants represents a core therapeutic strategy. Their advantages include precise matching of defect shapes, providing immediate mechanical support, and serving as templates for tissue regeneration [69]. Their application forms are primarily categorized into injectable hydrogels and 3D-printed scaffolds [70].

**Figure 7:**
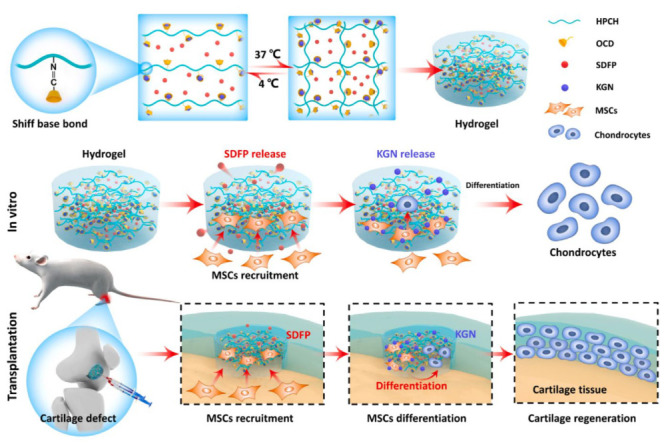
Schematic illustration of SDFP and KGN co-loaded HPCH hydrogel for stem cell recruitment and chondrogenic differentiation in cartilage regeneration. Reproduced with permission from ref. [73]. Copyright 2023, Elsevier.

Injectable PVA hydrogels have gained popularity due to their minimally invasive nature and conformability. These materials are typically injected as solutions into defect sites, where they rapidly gel under conditions such as body temperature, specific pH, or photoinitiation [71]. This type of gel exhibits shear-thinning behavior, facilitating smooth injection and post-injection self-healing. With a viscosity of <1000 Pa·s, it is suitable for injection and recovers to high viscosity at low shear rates, thereby resisting rapid clearance from the joint cavity [72]. In 2023, Yuan et al. [73] through in vitro experiments developed a dual-drug-loaded thermosensitive hydroxypropyl chitin (HPCH) hydrogel system, illustrated in Figure 7, through in vitro experiments for the sustained release of stromal cell-derived factor-1α-like peptide (SDFP) and kartogenin (KGN) to promote stem cell recruitment and chondrogenic differentiation. The HPCH/OCD-KGN200 hydrogel effectively promoted chondrogenic differentiation of stem cells and extracellular matrix secretion by chondrocytes. The incorporation of thermosensitive materials enables direct injection into specific joints, greatly simplifying surgical procedures and making the technique particularly suitable for minimally invasive procedures such as arthroscopy. Given that this formulation requires injection, we will subsequently consider using PVA incorporated into a thermosensitive in situ gel system as the base material, combining the tunability of PVA with the convenience of injectability afforded by thermosensitive materials.

3D-printed scaffolds fabricated with PVA-based bioinks enable the precise construction of three-dimensional structures with complex anatomical shapes, customized internal pore structures, and specific bioactivity distributions, based on patient-specific medical imaging data [74]. In 2015, Li et al. [75] utilized 3D printing technology to prepare PVA/silk fibroin composite scaffolds with biomimetic porous structures, incorporating TGF-β3 sustained-release microspheres within the pores. These pre-formed scaffolds not only provided stable three-dimensional support but also featured interconnected channels facilitating host cell migration, nutrient transport, and neovascularization of newly formed tissue. Compared with simple defect filling, these scaffolds with active induction functionality guided the regeneration of new cartilage, resulting in higher histological scores and better integration with surrounding cartilage. In 2022, P.B et al. [76] designed customized hierarchical meniscus scaffolds in SolidWorks and fabricated negative molds from polylactic acid (PLA) filaments via direct 3D printing. In 3D scaffolds seeded with MG63 osteosarcoma cells, significant increases in collagen secretion, cell proliferation, and biomechanical properties were observed, indicating suitability for cartilage tissue engineering. In 2024, Aitchison et al. [77] mixed alginate at different concentrations with polyvinyl alcohol (PVA) and fabricated hydrogel scaffolds using 3D bioprinting, followed by crosslinking with calcium chloride. By systematically adjusting the concentrations of PVA and alginate, the swelling behavior, degradation rate, and elastic modulus of the scaffolds could be effectively modulated. Among them, the composite scaffold containing 5% PVA and 20% alginate exhibited an elastic modulus of 0.22 MPa, which is close to that of natural cartilage. This strategy provides a bioink formulation with customizable physical and mechanical properties for cartilage tissue engineering, holding promise for biomedical applications such as cartilage repair.

The choice between injectable hydrogels and 3D-printed scaffolds depends on specific clinical requirements. Injectable hydrogels offer ease of operation and excellent defect conformity, though initial gel strength may be limited. 3D-printed scaffolds provide stable mechanical structures and flexible functional design, but implantation procedures may be more invasive. Future integration of both advantages—developing injectable “bioinks” capable of direct in situ printing during surgery to achieve personalized, high-precision defect repair—remains a prominent research focus.

### 3.3. Surface Replacement and Reconstruction

For extensive full-thickness cartilage wear resulting from osteoarthritis, local repair procedures are insufficient. In such cases, large-scale joint surface reconstruction becomes necessary. High-strength PVA-based hydrogels have demonstrated significant potential as artificial cartilage surface replacements in this field, aiming to partially or completely replace damaged articular cartilage surfaces and restore joint function [78]. This application imposes stringent requirements on substitute materials: they must exhibit extremely low friction coefficients, exceptional wear resistance, and creep resistance to withstand millions of cyclic loads over decades of joint use [79].

**Figure 8:**
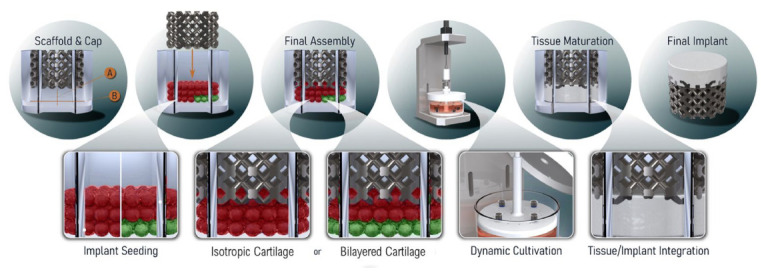
Biofabrication framework for the manufacture of cartilage-metal osteochondral implants. Reproduced with permission from ref. [81]. Copyright 2026, Elsevier.

In 2003, Kobayashi et al. [80] conducted a series of systematic studies evaluating the tribological and biomechanical properties of high-water-content PVA hydrogels as artificial cartilage surfaces for hip and knee joints. Their results demonstrated that high-water-content PVA-H is a promising implant material for reconstructing meniscal function and preventing osteoarthritic changes in articular cartilage, establishing a theoretical foundation for artificial cartilage applications. Achieving stable integration between artificial cartilage and the bone bed requires the development of osteochondral-integrated implants. This requires that the material itself, or the metal substrate, in combination with appropriate design, form a gradient transition from the superficial cartilage-replacement layer to the underlying osseointegration layer through the biofabrication framework shown in Figure 8 [81]. In 2014, Bichara et al. [82] synthesized PVA-poly(acrylic acid) (PAAc) hydrogel formulations and evaluated their efficacy in treating osteochondral defects in New Zealand white rabbit models. The PVA-PAAc hydrogels proved fully biocompatible, maintaining their properties over 12 weeks. Physical fixation of PVA-PAAc hydrogels to subchondral bone ensured long-term performance in repairing osteochondral defects. In 2024, Du et al. [83] fabricated porous polymer scaffolds with multi-scale porosity for bone tissue engineering using fused deposition modeling 3D printing. They employed “pile” PVA lattices with 250 μm or 500 μm struts, combined with emulsion templating and extractable 3D-printed negative molds. In vitro experiments demonstrated that osteosarcoma cells (MG63) penetrated and filled microchannel scaffolds more effectively. Similarly, bone-derived MSCs migrated upward from monolayer cultures into channel scaffolds, proliferated, and exhibited significantly higher osteogenic marker expression than in non-channel scaffolds.

Joint surface replacement and reconstruction represent the most challenging direction for clinical application of PVA hydrogels. The core issues extend far beyond the material itself: First, fixation technology—achieving strong, durable integration between large-area hydrogels and subchondral bone or metal backings to prevent loosening caused by interfacial micromotion and fluid penetration. Second, edge integration—ensuring smooth, stable transitions between implants and surrounding healthy cartilage to avoid edge stress concentration and secondary wear [84]. Third, long-term reliability—under the demanding environment of full joint motion—remains uncertain: whether materials can withstand decades of complex multiaxial stresses, wear, and aging. The relationship between material degradation patterns and joint lifespan requires further investigation [85]. These challenges will remain focal points for future research in joint surface replacement and reconstruction.

## 4. Challenges and Future Perspectives

This review systematically summarizes the research progress on poly(vinyl alcohol) (PVA)-based hydrogels for the treatment of osteoarthritis. PVA’s excellent hydrophilicity, tunable mechanical properties, and favorable biocompatibility make it an ideal platform for mimicking the natural cartilage matrix and serving as a carrier for cells and bioactive factors. Through strategies such as physical/chemical crosslinking, nanocomposite reinforcement, and biomimetic design, significant improvements in mechanical strength, lubricity, and biofunctionalization of PVA-based hydrogels have been achieved. These advances demonstrate broad application prospects, ranging from temporary filling to the induction of regeneration. However, several challenges remain to be addressed before clinical translation and fulfillment of complex dynamic joint environment requirements:

(1) Preparation and Clinical Translation: Although laboratory-scale preparation methods for PVA hydrogels (e.g., cyclic freezing-thawing, chemical crosslinking) are well established, significant challenges remain in transitioning from laboratory samples to standardized medical devices. Current research primarily focuses on optimizing material properties while overlooking the development of large-scale manufacturing processes. PVA hydrogel properties—including water content, pore size, and mechanical strength—are highly sensitive to preparation parameters (temperature, time, concentration). Minor process fluctuations can lead to considerable batch-to-batch variation, making it difficult to meet stringent medical device quality requirements. Future research should prioritize scalable, sterilizable manufacturing processes. Developing continuous, automated production equipment and establishing standardized sterilization validation protocols to transition from laboratory-scale to large-scale sterile production that is compliant with medical device standards are considered crucial for successful clinical translation.
(2) Beyond the scalability issues of manufacturing processes, the translation of PVA hydrogels also faces a series of specific regulatory and clinical integration challenges. The first is sterilization requirements: conventional sterilization methods, such as autoclaving, can destroy the hydrogel structure and drastically reduce its mechanical properties; ethylene oxide sterilization may introduce toxic residues; and high-dose gamma or electron-beam irradiation can cause chain scission of PVA molecules. Therefore, it is necessary to develop at low temperatures. These nondestructive sterilization protocols comply with medical device regulations, such as those for supercritical carbon dioxide sterilization and optimized irradiation doses, while establishing corresponding validation standards. The second challenge is implant fixation and integration: PVA cannot actively bind to host tissues, making long-term stable fixation difficult to achieve solely through mechanical press-fitting or tissue suturing. Early postoperative migration, dislodgement, or interfacial micromotion may occur, leading to repair failure. To address this, researchers are exploring surface modification (e.g., with dopamine), porous/gradient structures to promote tissue ingrowth, and bioresorbable fixation devices. Furthermore, from a regulatory perspective, as a long-term implant, PVA hydrogels require comprehensive data on biocompatibility, mechanical stability, and degradation behavior. There is currently a lack of dedicated product standards for cartilage repair hydrogels. Establishing in vitro performance testing methods and animal models that correlate with clinical endpoints is a critical bottleneck for obtaining regulatory approval.
(3) Performance Synergy: A major challenge in current research lies in achieving cartilage-like mechanical properties while maintaining efficient tissue regeneration capability within a single material system. To withstand joint loading, researchers have significantly enhanced the strength and wear resistance of PVA hydrogels using nanocomposite and double-network strategies. However, these dense networks often compromise porosity, hindering cell migration and nutrient diffusion and ultimately preventing regeneration. Conversely, porous scaffolds or growth factor-loaded soft gels designed to promote regeneration frequently lack sufficient mechanical properties to support early mobilization. Future modification strategies should aim for integrated and intelligent performance. Drawing inspiration from the gradient structure and anisotropy of natural cartilage, biomimetic gradient scaffolds can be designed using 3D/4D printing technologies. Developing multi-stimuli-responsive (e.g., pH- or enzyme-specific) drug- or growth factor-controlled release systems may achieve synergistic effects between mechanical protection and biological signal delivery during the repair process.
(4) To address the challenge of synergistic performance, it is necessary to move beyond the single-material paradigm toward multiphase, multifunctional integration. For example, constructing a “soft–hard” composite system: a dense PVA layer for load-bearing and a porous PVA layer to promote cell ingrowth. However, interfacial bonding between layers is a critical issue that can be improved through layer-by-layer freeze–thaw cycling or in situ graft polymerization. Furthermore, conventional homogeneous PVA hydrogels struggle to mimic the modulus of natural cartilage. Introducing reversible physical crosslinks (e.g., hydrogen bonds, coordination bonds) or double networks enables the hydrogel to dissipate energy under large deformation while maintaining integrity, achieving self-reinforcement or self-healing and enhancing long-term stability. Finally, in vitro mechanical testing should more closely replicate the real joint environment, shifting from static compression to multiaxial fatigue testing (including shear, torsion, and impact) to accurately evaluate the practical performance of the hydrogel.
(5) Osteoarthritis Treatment: PVA hydrogels in osteoarthritis therapy should function not merely as physical barriers or supports, but as bioactive platforms that modulate the local microenvironment and guide endogenous repair capabilities. Although studies incorporating growth factors or stem cells exist, research on maintaining their activity in complex inflammatory environments and achieving precise controlled release remains at a preliminary stage. Therefore, future PVA-based hydrogel therapeutic systems must possess immunomodulatory and microenvironment management capabilities. Designing smart hydrogels with anti-inflammatory, antioxidant (e.g., incorporating tannic acid), or immunomodulatory (e.g., guiding macrophage polarization toward the regenerative M2 phenotype) functions can actively create a regeneration-favorable immune microenvironment. Through such technological advances, PVA-based hydrogels are expected to transcend their current role as simple fillers and evolve into intelligent medical systems capable of sensing and actively regulating their environment.
(6) Beyond immunomodulation, the application of PVA hydrogels for osteoarthritis treatment should also consider the disease staging and combination therapy strategies. Osteoarthritis involves multiple pathological events, including cartilage degradation, synovial inflammation, and subchondral bone remodeling. An ideal hydrogel should address the entire disease process, for example, by co-delivering anti-degradation agents (e.g., MMP inhibitors) and pro-anabolic factors (e.g., TGF-β) to achieve a dual “protection–repair” function. Furthermore, drug release can be coupled with the mechanical environment of the joint: using piezoelectric materials or mechanosensitive molecular switches enables on-demand drug release triggered by cyclic pressure from movement. For large or irregular defects, injectable in situ forming hydrogels offer a minimally invasive approach to match individualized contours; however, challenges remain in post-curing mechanical strength and tissue integration. Finally, for composite hydrogels incorporating degradable components, the degradation rate and product safety must be systematically evaluated, and long-term simulation studies using animal models of osteoarthritis are required to verify their clinical superiority over existing treatment strategies.
(7) Building on 3D printing, 4D bioprinting, as a cutting-edge technology, introduces a dynamic regulatory dimension to cartilage regeneration by incorporating smart materials that can undergo morphological or functional changes over time in response to external stimuli (e.g., temperature, humidity, pH, or enzymatic activity). For example, it enables the printing of planar scaffolds that spontaneously curl into a meniscus shape, or microstructures that slowly unfold in the in vivo environment to adapt to defect cavities. Concurrently, artificial intelligence (AI)-assisted biomaterial design is revolutionizing scaffold development—by employing machine learning algorithms to analyze the correlations between material compositions, processing parameters, and biological outcomes, AI can efficiently predict and optimize bioink formulations and mechanical properties, thereby substantially shortening the translation cycle from laboratory to clinic. In the future, the deep integration of the dynamic deformation and spatiotemporal programming capabilities of 4D bioprinting, AI-driven rational material design, and the aforementioned strategies for biofunctionalization and degradation control is expected to construct truly intelligent and personalized cartilage regeneration systems.

## Figures and Tables

**Table 1:** Preparation methods for PVA-based hydrogels.

Strategy	Core Techniques	Limitations	Reference
Network Construction	Physical crosslinking, chemical crosslinking, radiation crosslinking	Potential residual toxicity from chemical methods; relatively loose single-network structure; limited mechanical properties.	[17,18,19,20,21,22,23,24,25,26,27]
Mechanical Enhancement	Interpenetrating networks (IPNs), dynamic bonds, biomimetic structures	Complex preparation processes; challenges in component compatibility; difficulty in scaling up biomimetic structures.	[28,29,30,31,32,33,34,35,36,37,38]
Biofunctionalization	Surface modification, controllable degradation, growth factor loading	Risk of bioactivity loss for loaded factors; difficulty in matching degradation rate with tissue regeneration; challenges in achieving long-term controlled release in vivo.	[39,40,41,42,43,44,45,46,47,48,49,50,51,52,53]

**Table 2:** Comparison of network construction methods.

Method	Principle	Limitations	Applications	Reference
Physical Crosslinking	Microcrystallites formed via repeated freezing-thawing cycles act as physical crosslinking points.	Limited mechanical strength; lower stability than chemically crosslinked networks.	Tissue engineering scaffolds; cartilage substitutes; cell/drug carriers.	[19,20,21]
Chemical Crosslinking	Covalent bonds are formed between crosslinking agents and hydroxyl groups on PVA chains.	Potential residual crosslinker toxicity; requires careful control of reaction conditions.	High-strength materials; absorbents; applications demanding superior mechanical performance.	[22,23]
Radiation Crosslinking	Free radicals generated by γ-rays or electron beams induce covalent crosslinking.	High equipment costs, precise dose control needed; challenges in mass production.	Medical dressings; cartilage repair; wound dressings; sterile implantable devices.	[24,25,26,27]

**Table 3:** Comparison of mechanical property enhancement strategies.

Preparation Method	Reaction Principle	Main Limitations	Application Fields	Reference
Interpenetrating Polymer Networks (IPN)	Two or more polymer networks interpenetrate and entangle.	Poor component compatibility; complex multi-step processing.	Cartilage repair; load-bearing tissue replacement.	[29,30,31]
Dynamic Reversible Bonds	Reversible breaking/reformation of metal coordination or hydrogen bonds enables self-healing.	Poor long-term stability; sensitive to environmental conditions.	Self-healing materials; flexible electronics.	[32,33,34]
Biomimetic Structural Design	Mimics nacre “brick-and-mortar” or wood anisotropic structures.	Difficult to scale up; complex structural control required.	Artificial cartilage/ligaments; tissue engineering scaffolds.	[35,36,37,38]

**Table 4:** Common biofunctionalization strategies.

Preparation Method	Reaction Principle	Main Limitations	Application Fields	Reference
Surface Modification	Introduction of coatings to improve cell adhesion and hemocompatibility.	Difficult to control coating stability and coverage uniformity.	Vascular grafts; cell scaffolds.	[40,41,42,43]
Introduction of Degradable Units	Incorporation of enzyme-cleavable peptide segments for controlled degradation.	Degradation rate mismatches with tissue regeneration rate.	Absorbable scaffolds; tissue engineering.	[44,45,46,47]
Growth Factor Loading	Integration of growth factors to promote localized regeneration.	Risk of factor inactivation; burst release effect.	Wound dressings; cartilage regeneration.	[48,49,50,51,52,53,54]

**Table 5:** Applications of PVA-based hydrogels in articular cartilage surgery.

Application Type	Representative Composition	Mechanical Properties	Primary Applications	Advantages	Key Challenges	Reference
Surgical Adjunctive Materials (preclinical)	PTGH + Icariin	Compressive strength: 2.87 ± 0.45 MPa; compressive modulus: 0.761 ± 0.072 MPa	Anti-adhesion membranes; drug/cell delivery vehicles.	Physical barrier preventing adhesions; localized, sustained release; dual barrier + therapy functionality.	Matching the degradation rate to the healing window to achieve long-term release of bioactive factors.	[58,59,60,61,62,63,64,65,66]
Replacement Implants (in vitro and preclinical)	3D-printed PVA/alginate scaffold	Elastic modulus: 0.22 MPa; tunable by adjusting the PVA/alginate ratio	Injectable thermosensitive gels; 3D-printed scaffolds.	Minimally invasive injection; mechanical stability; induction of stem cell differentiation.	Insufficient initial strength; invasive implantation for 3D-printed scaffolds.	[67,68,69,70,71,72,73,74]
Surface Replacement & Reconstruction (Preclinical)	PVA-poly(acrylic acid) (PAAc) hydrogel	Maintains stable mechanical properties for 12 weeks in the rabbit model; low friction, high wear resistance, creep resistance	High-strength PVA/PAAc; PVA/Nomex^®^ composites; osteochondral integrated implants.	Low friction coefficient; high wear resistance; creep resistance; long-term load bearing.	Poor long-term stability of interfacial fixation; difficulty achieving smooth edge integration.	[75,76,77,78,79,80,81,82]

## Data Availability

No new data were created or analyzed in this study. Data sharing is not applicable to this article.

## References

[1] Yao H., Kang J., Li W., Liu J., Xie R., Wang Y., Liu S., Wang D.A., Ren L. (2017). Novel β-TCP/PVA bilayered hydrogels with considerable physical and bio-functional properties for osteochondral repair. Biomed. Mater..

[2] Fox A.J.S., Bedi A., Rodeo S.A. (2009). The Basic Science of Articular Cartilage: Structure, Composition, and Function. Sports Health.

[3] Vilá y Rico J., Dalmau A., Chaqués F.J., Asunción J. (2015). Treatment of Osteochondral Lesions of the Talus With Bone Marrow Stimulation and Chitosan–Glycerol Phosphate/Blood Implants (BST-CarGel). Arthrosc. Tech..

[4] Knutsen G., Engebretsen L., Ludvigsen T.C., Drogset J.O., Grøntvedt T., Solheim E., Strand T., Roberts S., Isaksen V., Johansen O. (2004). Autologous Chondrocyte Implantation Compared with Microfracture in the Knee: A Randomized Trial. J. Bone Jt. Surg..

[5] Scholten P.M., Ng K.W., Joh K., Serino L.P., Warren R.F., Torzilli P.A., Maher S.A. (2011). A semi-degradable composite scaffold for articular cartilage defects. J. Biomed. Mater. Res. Part A.

[6] Li J., Mooney D.J. (2016). Designing hydrogels for controlled drug delivery. Nat. Rev. Mater..

[7] Drury J.L., Mooney D.J. (2003). Hydrogels for tissue engineering: Scaffold design variables and applications. Biomaterials.

[8] Di J., Li J., Sun C., Xu L., Li X. (2025). Advances in Cellulose-Based Hydrogels for Drug Delivery: Preparation, Modification and Challenges. Gels.

[9] Burdick J.A., Mauck R.L., Gorman J.H., Gorman R.C. (2013). Acellular biomaterials: An evolving alternative to cell-based therapies. Sci. Transl. Med..

[10] Elisseeff J., Puleo C., Yang F., Sharma B. (2005). Advances in skeletal tissue engineering with hydrogels. Orthod. Craniofacial Res..

[11] Hashemi-Afzal F., Fallahi H., Bagheri F., Collins M.N., Eslaminejad M.B., Seitz H. (2025). Advancements in hydrogel design for articular cartilage regeneration: A comprehensive review. Bioact. Mater..

[12] Noguchi T., Yamamuro T., Oka M., Kumar P., Kotoura Y., Hyonyt S.-H., Ikadat Y. (1991). Poly(vinyl alcohol) hydrogel as an artificial articular cartilage: Evaluation of biocompatibility. J. Appl. Biomater..

[13] Kobayashi M., Hyu H.S. (2010). Development and Evaluation of Polyvinyl Alcohol-Hydrogels as an Artificial Atrticular Cartilage for Orthopedic Implants. Materials.

[14] Rahman Khan M.M., Rumon M.M.H. (2025). Synthesis of PVA-Based Hydrogels for Biomedical Applications: Recent Trends and Advances. Gels.

[15] Leone G., Consumi M., Pepi S., Pardini A., Bonechi C., Tamasi G., Donati A., Lamponi S., Rossi C., Magnani A. (2020). Enriched Gellan Gum hydrogel as visco-supplement. Carbohydr. Polym..

[16] Todros S., Barbon S., Stocco E., Favaron M., Macchi V., De Caro R., Porzionato A., Pavan P.G. (2022). Time-dependent mechanical behavior of partially oxidized polyvinyl alcohol hydrogels for tissue engineering. J. Mech. Behav. Biomed. Mater..

[17] Roca-Arroyo A.F., Gutierrez-Rivera J.A., Morton L.D., Castilla-Casadiego D.A. (2025). Hydrogel Network Architecture Design Space: Impact on Mechanical and Viscoelastic Properties. Gels.

[18] Hoffman A.S. (2012). Hydrogels for biomedical applications. Adv. Drug Deliv. Rev..

[19] Adelnia H., Ensandoost R., Shebbrin Moonshi S., Gavgani J.N., Vasafi E.I., Ta H.T. (2022). Freeze/thawed polyvinyl alcohol hydrogels: Present, past and future. Eur. Polym. J..

[20] Górska A., Baran E., Knapik-Kowalczuk J., Szafraniec-Szczęsny J., Paluch M., Kulinowski P., Mendyk A. (2024). Physically Cross-Linked PVA Hydrogels as Potential Wound Dressings: How Freezing Conditions and Formulation Composition Define Cryogel Structure and Performance. Pharmaceutics.

[21] Hassan C.M., Peppas N.A. (2000). Structure and Applications of Poly(vinyl alcohol) Hydrogels Produced by Conventional Crosslinking or by Freezing/Thawing Methods. Biopolymers · PVA Hydrogels, Anionic Polymerisation Nanocomposites.

[22] Kudo K., Ishida J., Syuu G., Sekine Y., Ikeda-Fukazawa T. (2014). Structural changes of water in poly(vinyl alcohol) hydrogel during dehydration. J. Chem. Phys..

[23] Al-Sabagh A.M., Abdeen Z. (2010). Preparation and Characterization of Hydrogel Based on Poly(vinyl alcohol) Cross-Linked by Different Cross-Linkers Used to Dry Organic Solvents. J. Polym. Environ..

[24] Jin S.G. (2022). Production and Application of Biomaterials Based on Polyvinyl alcohol (PVA) as Wound Dressing. Chem. Asian J..

[25] Rosiak J.M., Ulański P. (1999). Synthesis of hydrogels by irradiation of polymers in aqueous solution. Radiat. Phys. Chem..

[26] Chen Y., Yang M., Zhang W., Guo W., Zhang X., Zhang B. (2024). Facile Preparation of Irradiated Poly(vinyl alcohol)/Cellulose Nanofiber Hydrogels with Ultrahigh Mechanical Properties for Artificial Joint Cartilage. Materials.

[27] Khalid M., Jameel F., Jabri T., Jabbar A., Salim A., Khan I., Shah M.R. (2024). α-Terpineol loaded, electron beam crosslinked polyvinyl alcohol/tapioca starch hydrogel sheets; fabrication, characterization and evaluation of wound healing potential on a full thickness acid burn wound. RSC Adv..

[28] Wang Y.-Q., Zhu Y., Wang J.-H., Li X.-N., Wu X.-G., Qin Y.-X., Chen W.-Y. (2021). Fe3+, NIR light and thermal responsive triple network composite hydrogel with multi-shape memory effect. Compos. Sci. Technol..

[29] Gong J.P., Katsuyama Y., Kurokawa T., Osada Y. (2003). Double-Network Hydrogels with Extremely High Mechanical Strength. Adv. Mater..

[30] Pulat M., Özgündüz H.İ. (2014). Swelling behavior and morphological properties of semi-IPN hydrogels based on ionic and non-ionic components. Bio-Med. Mater. Eng..

[31] Santos F., Marto-Costa C., Branco A.C., Oliveira A.S., Galhano dos Santos R., Salema-Oom M., Diaz R.L., Williams S., Colaço R., Figueiredo-Pina C. (2024). Tribomechanical Properties of PVA/Nomex^®^ Composite Hydrogels for Articular Cartilage Repair. Gels.

[32] Sun J.-Y., Zhao X., Illeperuma W.R.K., Chaudhuri O., Oh K.H., Mooney D.J., Vlassak J.J., Suo Z. (2012). Highly stretchable and tough hydrogels. Nature.

[33] Fan H., Wang J., Zhang Q., Jin Z. (2017). Tannic Acid-Based Multifunctional Hydrogels with Facile Adjustable Adhesion and Cohesion Contributed by Polyphenol Supramolecular Chemistry. ACS Omega.

[34] Zhang W., Kuss M., Yan Y., Shi W. (2023). Dynamic Alginate Hydrogel as an Antioxidative Bioink for Bioprinting. Gels.

[35] Guan Q.-F., Ling Z.-C., Han Z.-M., Yang H.-B., Yu S.-H. (2020). Ultra-Strong, Ultra-Tough, Transparent, and Sustainable Nanocomposite Films for Plastic Substitute. Matter.

[36] Ji D., Zhang Z., Sun J., Cao W., Wang Z., Wang X., Cao T., Han J., Zhu J. (2024). Strong, Tough, and Biocompatible Poly(vinyl alcohol)–Poly(vinylpyrrolidone) Multiscale Network Hydrogels Reinforced by Aramid Nanofibers. ACS Appl. Mater. Interfaces.

[37] Mredha M.T.I., Jeon I. (2022). Biomimetic anisotropic hydrogels: Advanced fabrication strategies, extraordinary functionalities, and broad applications. Prog. Mater. Sci..

[38] Soto-Quintero A., González Castillo E.I., Gómez Lizárraga K., Barba-Pingarrón A., Hernández M. (2024). Enhancing the performance of nanostructured PVA/SA scaffolds through incorporation of macromolecules: From synergistic effects to advanced multifunctionalities. Mater. Lett..

[39] Cavallo C., Amore E., Carpentieri S., Roseti L. (2026). Challenges and Strategies in Hydrogel-Based Cartilage Regeneration. Gels.

[40] Nie L., Zhang G., Hou R., Xu H., Li Y., Fu J. (2015). Controllable promotion of chondrocyte adhesion and growth on PVA hydrogels by controlled release of TGF-β1 from porous PLGA microspheres. Colloids Surf. B Biointerfaces.

[41] Hou R., Nie L., Du G., Xiong X., Fu J. (2015). Natural polysaccharides promote chondrocyte adhesion and proliferation on magnetic nanoparticle/PVA composite hydrogels. Colloids Surf. B Biointerfaces.

[42] Oliveira A.S., Schweizer S., Nolasco P., Barahona I., Saraiva J., Colaço R., Serro A.P. (2020). Tough and Low Friction Polyvinyl Alcohol Hydrogels Loaded with Anti-inflammatories for Cartilage Replacement. Lubricants.

[43] Barbon S., Confalonieri M., Stocco E., D’Osualdo A., Contran M., Parnigotto P.P., De Caro R., Todros S., Macchi V., Pavan P.G. (2025). Bio-hybrid scaffolds combining polyvinyl alcohol and decellularized articular cartilage for the treatment of focal chondral lesions in hemophilic patients. Front. Pharmacol..

[44] Wu Y., Yu C., Xing M., Wang L., Guan G. (2020). Surface modification of polyvinyl alcohol (PVA)/polyacrylamide (PAAm) hydrogels with polydopamine and REDV for improved applicability. J. Biomed. Mater. Res. Part B Appl. Biomater..

[45] Xie Y., Liu Q., Tan J., Lu Y., Li Y., Ye H., Xu Z., Wang M., Wang C., Li X. (2026). CAP-CD56(+)CD271(+) BMSCs exos-loaded PVA/SA sustained-release hydrogel attenuates chondrocyte senescence and ameliorates lumbar facet joint osteoarthritis. Bioact. Mater..

[46] Liu Y., Meng H., Konst S., Sarmiento R., Rajachar R., Lee B.P. (2014). Injectable Dopamine-Modified Poly(ethylene glycol) Nanocomposite Hydrogel with Enhanced Adhesive Property and Bioactivity. ACS Appl. Mater. Interfaces.

[47] Li J., Wang Z., Yang W., Zhang Y., Wang Y., Wang X., Wang H., Xie Y., Xu S., Shang Y. (2026). Bionic Janus hydrogel drives infected Achilles tendon regeneration via mechano-immune spatiotemporal steering. Nat. Commun..

[48] Saidin S., Zubairi S.I., Ambreen J., Md Lazim N.A., Sadia M., Lim J.T.W., Ulum M.F., Elliyanti A., Sefat F. (2026). Biode-gradable synthetic polymers for biomedical and tissue engineering applications: Tailoring degradation kinetics with tissue regeneration timeline. BioMed. Eng. OnLine.

[49] Bastard C., Schulte J.C., Asaduzzaman M., Hohn C., Kittel Y., De Laporte L., Gebhardt R. (2025). Casein microparticles filled with cellulase to enzymatically degrade nanocellulose for cell growth. Biomater. Sci..

[50] Cai Q., Yang J., Bei J., Wang S. (2002). A novel porous cells scaffold made of polylactide–dextran blend by combining phase-separation and particle-leaching techniques. Biomaterials.

[51] Deller R.C., Richardson T., Richardson R., Bevan L., Zampetakis I., Scarpa F., Perriman A.W. (2019). Artificial cell membrane binding thrombin constructs drive in situ fibrin hydrogel formation. Nat. Commun..

[52] Thai N.L.B., Beaman H.T., Perlman M., Obeng E.E., Du C., Monroe M.B.B. (2024). Chitosan Poly(vinyl alcohol) Methacrylate Hydrogels for Tissue Engineering Scaffolds. ACS Appl. Bio Mater..

[53] Hu M., Tang Y., He X., Liu K., Qin L., Wang X., Wang Q. (2026). Enzyme-Integrated Hydrogels for Advanced Biological Applications. Polym. Sci. Technol..

[54] Li Y., Sang Z., Chen S., Li H., Ren X., Mei X., Chen Z. (2025). A rationally engineered PVA-HA hydrogel orchestrates osteochondral regeneration and matrix mineralization by cytoprotecting chondrocytes mitochondrion. Chem. Eng. J..

[55] Yasuda K., Ping Gong J., Katsuyama Y., Nakayama A., Tanabe Y., Kondo E., Ueno M., Osada Y. (2005). Biomechanical properties of high-toughness double network hydrogels. Biomaterials.

[56] Spiller K.L., Holloway J.L., Gribb M.E., Lowman A.M. (2011). Design of semi-degradable hydrogels based on poly(vinyl alcohol) and poly(lactic-co-glycolic acid) for cartilage tissue engineering. J. Tissue Eng. Regen. Med..

[57] Gong J.P., Kurokawa T., Narita T., Kagata G., Osada Y., Nishimura G., Kinjo M. (2001). Synthesis of hydrogels with extremely low surface friction. J. Am. Chem. Soc..

[58] ten Broek R.P.G., Strik C., Issa Y., Bleichrodt R.P., van Goor H. (2013). Adhesiolysis-Related Morbidity in Abdominal Surgery. Ann. Surg..

[59] Liu Y., Li H., Shu X.Z., Gray S.D., Prestwich G.D. (2005). Crosslinked hyaluronan hydrogels containing mitomycin C reduce postoperative abdominal adhesions. Fertil. Steril..

[60] Bae S.-H., Son S.-R., Kumar Sakar S., Nguyen T.-H., Kim S.-W., Min Y.-K., Lee B.-T. (2014). Evaluation of the potential anti-adhesion effect of the PVA/Gelatin membrane. J. Biomed. Mater. Res. Part B Appl. Biomater..

[61] Ma P., Liang W., Huang R., Zheng B., Feng K., He W., Huang Z., Shen H., Wang H., Wu D. (2024). Super-Structured Wet-Adhesive Hydrogel with Ultralow Swelling, Ultrahigh Burst Pressure Tolerance, and Anti-Postoperative Adhesion Properties for Tissue Adhesion. Adv. Mater..

[62] Lee H., Jeong Y., Lee N., Lee I., Lee J.H. (2025). Recent Advances in Injectable Hydrogels for Biomedical and Aesthetic Applications: Focus on Rheological Characteristics. Gels.

[63] Hunziker E.B. (2002). Articular cartilage repair: Basic science and clinical progress. A review of the current status and prospects. Osteoarthr. Cartil..

[64] Fenton O.S., Olafson K.N., Pillai P.S., Mitchell M.J., Langer R. (2018). Advances in Biomaterials for Drug Delivery. Adv. Mater..

[65] Spiller K.L., Liu Y., Holloway J.L., Maher S.A., Cao Y., Liu W., Zhou G., Lowman A.M. (2012). A novel method for the direct fabrication of growth factor-loaded microspheres within porous nondegradable hydrogels: Controlled release for cartilage tissue engineering. J. Control. Release.

[66] Dashtdar H., Murali M.R., Abbas A.A., Suhaeb A.M., Selvaratnam L., Tay L.X., Kamarul T. (2015). PVA-chitosan composite hydrogel versus alginate beads as a potential mesenchymal stem cell carrier for the treatment of focal cartilage defects. Knee Surg. Sports Traumatol. Arthrosc..

[67] Xiang C., Guo Z., Zhang Q., Wang Z., Li X., Chen W., Wei X., Li P., Xiang C. (2024). Physically crosslinked poly(vinyl alcohol)-based hydrogels for cartilage tissue engineering. Mater. Des..

[68] Chelu M., Musuc A.M. (2023). Polymer Gels: Classification and Recent Developments in Biomedical Applications. Gels.

[69] Cercone M., Chevalier J., Kennedy J.G., Miller A.D., Fortier L.A. (2021). Early Failure of a Polyvinyl Alcohol Hydrogel Implant with Osteolysis and Foreign Body Reactions in an Ovine Model of Cartilage Repair. Am. J. Sports Med..

[70] Alcala-Orozco C.R., Mutreja I., Cui X., Hooper G.J., Lim K.S., Woodfield T.B.F. (2022). Hybrid biofabrication of 3D osteoconductive constructs comprising Mg-based nanocomposites and cell-laden bioinks for bone repair. Bone.

[71] Ghandforoushan P., Alehosseini M., Golafshan N., Castilho M., Dolatshahi-Pirouz A., Hanaee J., Davaran S., Orive G. (2023). Injectable hydrogels for cartilage and bone tissue regeneration: A review. Int. J. Biol. Macromol..

[72] von Lospichl B., Hemmati-Sadeghi S., Dey P., Dehne T., Haag R., Sittinger M., Ringe J., Gradzielski M. (2017). Injectable hydrogels for treatment of osteoarthritis—A rheological study. Colloids Surf. B Biointerfaces.

[73] Yuan X., Wan J., Yang Y., Huang L., Zhou C., Su J., Hua S., Pu H., Zou Y., Zhu H. (2023). Thermosensitive hydrogel for cartilage regeneration via synergistic delivery of SDF-1α like polypeptides and kartogenin. Carbohydr. Polym..

[74] Hong S., Sycks D., Chan H.F., Lin S., Lopez G.P., Guilak F., Leong K.W., Zhao X. (2015). 3D Printing of Highly Stretchable and Tough Hydrogels into Complex, Cellularized Structures. Adv. Mater..

[75] Li X., Qin J., Ma J. (2015). Silk fibroin/poly (vinyl alcohol) blend scaffolds for controlled delivery of curcumin. Regen. Biomater..

[76] Sathish P.B., Gayathri S., Priyanka J., Shalini M., Narmadha R., Shankar K.G., Selvakumar R. (2022). Tricomposite gelatin-carboxymethylcellulose-alginate bioink for direct and indirect 3D printing of human knee meniscal scaffold. Int. J. Biol. Macromol..

[77] Aitchison A.H., Allen N.B., Mitra K., Abar B., O’Neill C.N., Bagheri K., Anastasio A.T., Adams S.B. (2024). Tunable Alginate-Polyvinyl Alcohol Bioinks for 3D Printing in Cartilage Tissue Engineering. Gels.

[78] Zhong Y., Lin Q., Yu H., Shao L., Cui X., Pang Q., Zhu Y., Hou R. (2024). Construction methods and biomedical applications of PVA-based hydrogels. Front. Chem..

[79] Hu D., Yan Y., Wei W., Bai C., Lu Y., Wang Y., Zhai F., Liu D., Wang X. (2025). Mechanically Robust Lubricating Hydrogels Contrived by Harnessing Low-Entropy Nanocrystalline Polymer Network. Adv. Funct. Mater..

[80] Kobayashi M., Toguchida J., Oka M. (2003). Preliminary study of polyvinyl alcohol-hydrogel (PVA-H) artificial meniscus. Biomaterials.

[81] Kotlarz M., O’Keeffe C., Kronemberger G.S., Burdis R., Rana S., Rodriguez N., Bonizzi M., Brama P., Kelly D.J. (2026). Biofabrication and in vivo evaluation of a hybrid osteochondral implant consisting of structurally organised engineered cartilage on a 3D-printed metal scaffold. Biomaterials.

[82] Bichara D.A., Bodugoz-Sentruk H., Ling D., Malchau E., Bragdon C.R., Muratoglu O.K. (2014). Osteochondral defect repair using a polyvinyl alcohol-polyacrylic acid (PVA-PAAc) hydrogel. Biomed. Mater..

[83] Du S., Huynh T., Lu Y.-Z., Parker B.J., Tham S.K., Thissen H., Martino M.M., Cameron N.R. (2024). Bioactive polymer composite scaffolds fabricated from 3D printed negative molds enable bone formation and vascularization. Acta Biomater..

[84] Sun H., Zhao Y. (2025). Bionic Peek with Drug-Loaded Hydrogel for Cartilage Repair. Int. Dent. J..

[85] Yin C., Huang Z., Zhang Y., Ren K., Liu S., Luo H., Zhang Q., Wan Y. (2024). Strong, tough, and elastic poly(vinyl alcohol)/polyacrylamide DN hydrogels based on the Hofmeister effect for articular cartilage replacement. J. Mater. Chem. B.

